# Sarcoidosis with involvement of the paranasal sinuses - a retrospective analysis of 12 biopsy-proven cases

**DOI:** 10.1186/1471-2466-13-59

**Published:** 2013-09-26

**Authors:** Anne-Marie Kirsten, Henrik Watz, Detlef Kirsten

**Affiliations:** 1 Pulmonary Research Institute at LungClinic Grosshansdorf, Airway Research Centre North, Member of the German Center for Lung Research, Grosshansdorf, Germany; 2 Outpatient Clinic for Rare Lung Diseases and Sarcoidosis at LungClinic Grosshansdorf, Airway Research Center North, Member of the German Center for Lung Research, Grosshansdorf, Germany

**Keywords:** Sarcoidosis, Sinonasal involvement, Treatment of sarcoidosis

## Abstract

**Background:**

Extrapulmonary involvement by sarcoidosis is observed in about 30–40% of patients with sarcoidosis. Little is known about the frequency and clinical characteristics of sinonasal sarcoidosis.

**Methods:**

We retrospectively analyzed 12 cases of biopsy-proven sinonasal sarcoidosis. Patients were identified from a patient population of 1360 patients with sarcoidosis at the Outpatient Clinic for Sarcoidosis and Rare Lung Diseases at LungClinic Grosshansdorf, a tertiary care hospital for respiratory medicine.

**Results:**

The most frequent signs and symptoms were nasal polyps (4 cases), epistaxis (3 cases), nasal crusts (8 cases) and anosmia (5 cases). Pulmonary sarcoidosis of the patients was staged as stage I (n = 1) and stage II (n = 11) on chest radiographs. Spirometry was normal in 11 patients. 7 patients had a diffusion capacity of the lung for carbon monoxide of less than 90% of predicted. Other organs were affected in 8 patients. All patients received systemic corticosteroid treatment and most patients received topical steroids. 5 patients received steroid sparing agents. Repeated sinus surgery had to be performed in 4 patients.

**Conclusions:**

Sinonasal involvement is a rare disease manifestation of sarcoidosis with a frequency slightly lower than 1% in our patient population. The clinical course of sinonasal sarcoidosis can be complicated by relapse despite systemic immunosuppressive treatment and repeated sinus surgery.

## Background

Sarcoidosis is a multisystem granulomatous disease commonly involving the lungs and the mediastinum. Sarcoidosis may not only affect the lungs and mediastinum but also any other organ and extrapulmonary involvement is observed in about 30–40% of patients with sarcoidosis [1].

The incidence of sarcoidosis in Germany has been estimated by Scharkoff with about 10/100.000 per year [2]. A survey among German patients with sarcoidosis organized in the German Sarcoidosis self-help group revealed a frequency of extrapulmonary involvement by sarcoidosis of 47.5% (but histologically proven in only 30%) [3].

Even though predilection of the respiratory tract by sarcoidosis and extrapulmonary involvement of other organs is frequently observed there is only limited data available regarding the frequency of sinonasal involvement [4]. As the symptoms of patients with sinonasal sarcoidosis resemble the symptoms of chronic rhinitis and chronic inflammatory rhinosinusitis like nasal obstruction, epistaxis, rhinorrhea, nasal crusts, anosmia or facial pain sinonasal sarcoidosis might be an overlooked disease manifestation [5].

In 1983 McCaffrey and McDonald reviewed the records of 2319 patients with sarcoidosis to determine the incidence of nasal involvement. 17 patients were found to have nasal lesions proven by biopsy to be of non-caseating granulomatous inflammation [6].

Until now there are only very few data available regarding frequency and clinical characteristics of sinonasal sarcoidosis [7-13]. Here we report on 12 patients with biopsy-proven sinonasal sarcoidosis who were treated in a tertiary respiratory care setting.

## Methods

### Study population

We retrospectively analyzed 12 cases of biopsy-proven sinonasal sarcoidosis. Patients were identified at the Outpatient Clinic for Sarcoidosis and Rare Lung Diseases at LungClinic Grosshansdorf, a tertiary care hospital for respiratory medicine between 2007 and 2011. In total, 1360 patients with sarcoidosis were treated between 2007 and 2011, among these patients we observed about 500 patients with extrapulmonary organ involvement. 24 patients were identified by the treating pulmonologist (D.K.) to present with symptoms of sinonasal disease. Only 12 of these 24 patients had a sinonasal biopsy taken for histopathological confirmation of granulomatous inflammation and were included in our retrospective analysis. The histopathological confirmation in 12 patients was not performed due to adequate response to topical steroid treatment, mild symptoms, or because patients refused ENT surgery. 4 out of 12 patients had a coincidence of outset of their sarcoidosis and sinonasal involvement. In the remaining 8 patients there was a period of time of 1–15 years between the initial diagnosis of sarcoidosis and sinonasal manifestation.

According to German law an ethic approval was not necessary due to the retrospective design of our investigation and pseudonymization of patient data.

## Results

Among the 12 patients with sinonasal sarcoidosis, 4 were women and 8 were men. The mean age was 41 years (Table 1). All patients were Caucasians. The most frequent signs and symptoms were nasal polyps (4 cases), epistaxis (3 cases), nasal crusts (8 cases) and anosmia (5 cases, Table 2).

**Table 1:** Demographics of the 12 patients

**n**	**12**
Age, years mean (range)	41.1 (27–63)
Male/female	8/4
Löfgren’s syndrome	0
Pulmonary involvement	12
ACE (U/Liter) mean of all subjects (range)	72.9 (18–240)

**Table 2:** Clinical characteristics of 12 patients with biopsy proven sinonasal sarcoidosis

**#**	**Pulmonary involvement**	**Lymphe node involvement**	**Other organ involvement**	**CT scan of sinuses**	**Sinus involvement**	**Granulomatous inflammation at sinonasal biopsies**	Chest-X-ray type*	**Epistaxis**	**Anosmia**	**Crusting**	**Polyps**	FVC% pred**	DLCO% pred**
1	yes	no	bone	yes	MSB	yes	2	no	no	no	no	99.0	91.0
2	yes	no	CNS	yes	MSB, ES, FS	yes	2	no	no	yes	no	115.0	68.0
3	yes	yes	spleen, liver	yes	MSB, ES, FS	yes	1	yes	yes	yes	yes	97.3	100.8
4	yes	yes	none	yes	MSB, ES, FS	yes	2	no	yes	yes	no	76.6	64.6
5	yes	yes	none	yes	MSB	yes	2	no	no	no	yes	109.0	90.0
6	yes	yes	none	yes	MSS, ES, FS	yes	2	no	no	no	no	125.9	101.1
7	yes	yes	none	yes	MSB, ES	yes	2	n.a.	n.a.	n.a.	n.a.	89.5	67.3
8	yes	yes	bone, kidney, spleen	yes	MSB, ES, FS	yes	2	yes	yes	yes	no	126.0	55.0
9	yes	yes	bone	yes	ES, FS	yes	2	no	yes	yes	yes	133.0	90.6
10	yes	no	skin	n.a.	n.a.	yes	2	yes	yes	yes	no	50.0	63.0
11	yes	yes	skin	yes	MSB	yes	2	no	no	yes	yes	87.6	59.4
12	yes	yes	spleen, liver	yes	MSB, ES, FS	yes	2	no	no	yes	no	80.7	66.5

N.A., not available; CT, computed tomography; * according to Scadding [reference [14]; FVC, forced vital capacity; DLCO, diffusion capacity for carbon monoxide; ** predicted values according to European Community for Coal and Steel [reference [20,21]; MSB, maxillary sinus bilateral; MSS, maxillary sinus singular; ES, ethmoidal sinus; FS, frontal sinus.

The levels of the angiotensin-converting enzyme (ACE) in serum were elevated in 6 patients (> 52 Units/Liter). The mean value of ACE in all patients was 72.9 Units/Liter (Table 1).

Pulmonary sarcoidosis of the patients was staged according to chest X-rays based on the Scadding criteria [14] as stage/group I (n = 1) and stage/group II (n = 11; Table 2). Pulmonary biopsies (bronchial mucosa or transbronchial biopsy) revealed pulmonary involvement of sarcoidosis in all patients. In addition to sinonasal and pulmonary involvement sarcoidosis affected other organs in 8 patients (Table 2).

Spirometry was normal in the majority of our patients. Only one patient had a combined restrictive-obstructive ventilation abnormality (pulmonary involvement plus marked endobronchial mucosal hypertrophy) resulting in a forced vital capacity of 50% predicted (Table 2). 7 patients had a diffusion capacity of the lung for carbon monoxide (DLCO) of less than 90% of predicted (Table 2).

**Figure 1:**
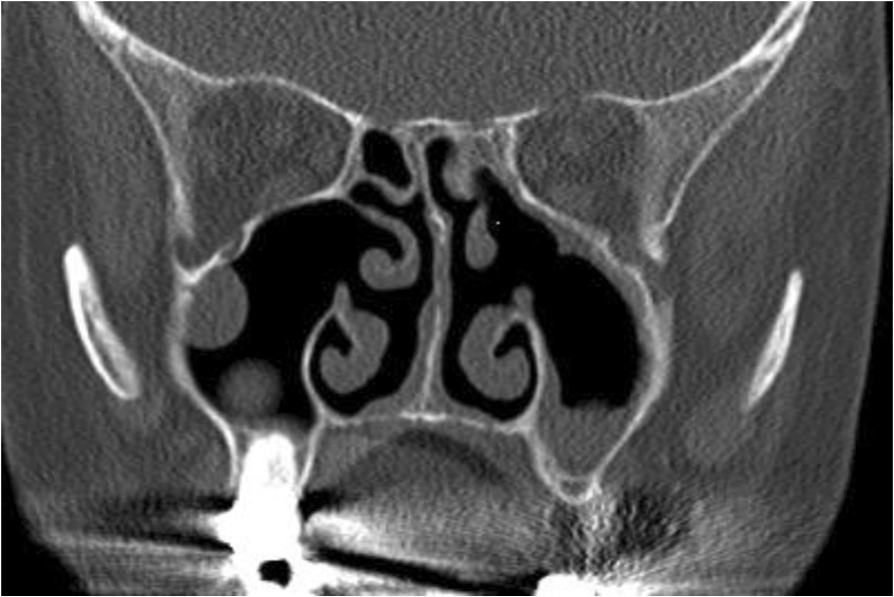
CT scan (coronal) of biopsy-proven sinonasal sarcoidosis affecting both maxillary sinuses.

None of the patients showed signs or symptoms of acute Sarcoidosis (Löfgren’s syndrome; Table 1). CT scan of the paranasal sinuses in 11 of 12 patients showed signs of chronic sinus inflammation with mucosal thickening and opacification of one or more sinuses (Figure 1). The involvement of the maxillary sinuses was more frequent than the involvement of the ethmoidal sinus or frontal sinus in our patients (Table 2).

The systematic review of the histopathological reports of the tissue biopsies revealed typical granulomatous inflammation in all cases (Table 2). In addition, in one patient the biopsies revealed eosinophiIic granulocytes being sporadically present in sinonasal tissue without any evidence of atopy or allergic asthma.

### Treatment

Sinus surgery was performed in all patients. The main reason for sinus surgery was histological confirmation of the suspected paranasal sinus involvement of sarcoidosis. In 4 patients sinus surgery had to be repeated - up to 6 times in one patient - because of persistent symptoms despite systemic corticosteroid treatment.

Medical treatment with systemic (oral) corticosteroids was initiated in all patients. Mean duration of corticosteroid treatment was 3 years (0.25-9 years). In addition, most patients received topical, intranasal steroids and 5 patients received steroid sparing agents like azathioprine, metotrexate, leflunomide or chloroquine. The indication and the duration of systemic corticosteroid treatment as well as the addition of steroid sparing agents were not always triggered by sinonasal symptoms only, but also by other organ involvements.

## Discussion

Interestingly, the nasal mucosa involvement of sarcoidosis is known since Boeck in 1905 (cited by Scadding [15]). Furthermore, involvement of nasal sinuses has been described by Schaumann in 1924 and 1926 (cited by Boettger [16]). However, it is currently difficult to estimate the exact prevalence of sinonasal involvement in patients with sarcoidosis. We found 12 patients out of 1360 patients with sarcoidosis to have biopsy proven sinonasal sarcoidosis. This frequency of slightly less than one percent of patients being affected by sinonasal involvement is in line with the reported frequency by McCaffrey and McDonald [6]. The exact prevalence of sinonasal involvement, however, might be clearly underestimated in patients with sarcoidosis as histopathological examination of sinonasal tissue is not regularly performed after sinonasal surgery [17]. We found in total 24 patients with symptoms of sinonasal disease in our study population. The frequency of sarcoidosis of the upper respiratory tractis estimated to be about 5% of patients with established diagnosis of sarcoidosis, although upper airway symptoms are more common in patients with sarcoidosis [4,13]. Therefore, the incidence of sinonasal involvement in sarcoidosis is generally believed to be clearly underestimated [17]. Table 3 summarizes the reported frequencies and clinical characteristics of patients with biopsy-proven sinonasal sarcoidosis as reported by sarcoidosis clinics and respiratory departments of tertiary care hospitals. It is difficult to find common characteristics of these studies, but is seems that the reported frequencies of sinonasal sarcoidosis are higher among study populations that involved African Americans.

**Table 3:** Frequency of biopsy-proven sinonasal sarcoidosis reported by sarcoidosis clinics/respiratory departments of tertiary care hospitals

**Authors, reference**	**Year**	**Study design**	**Country**	**Number of patients with sarcoidosis**	**Number of patients with biopsy-proven sinonasal sarcoidosis**	**Frequency of patients with sinonasal sarcoidosis**	**Race of patients with sinonasal sarcoidosis**	**Characteristics of patients with sinonasal sarcoidosis**
Wilson et al. ref. [12]	1988	Prospective	England	750	21	2.8%	44% Caucasian*^1^, 66% West Indian	Mean age, 41 years*^1^, Female, 52%, Lung involvement, 67%, Multiorgan involvement, n.a.
Zeitlin et al. ref. [11]	2000	Prospective (part I)	United States	159	6	3.8%	78% African Americans, 22% Caucasians	Mean age, 38 years, Female, 67%, Lung involvement, 72%, Multiorgan involvement, 89%
		Retrospective (Part II)		733	12	1.6%		
Panselinas et al. ref. [13]	2010	Retrospective	United States	998	43	4.3%	68% African*^2^ American, 31% Caucasian	Mean age, 38 years*^2^, Female, 75% Lung involvement, n.a., Multiorgan involvement, n.a.
Kirsten A et al. present study	2013	Retrospective	Germany	1360	12	0.8%	100% Caucasians	Mean age, 41 years, Female, 33%, Lung involvement, 100%, Multiorgan involvement, 67%

*^1^ race and characteristics refer to the whole population including 6 patients with probable sinonasal sarcoidosis (i.e. no histology available).

*^2^ race and characteristics refer to the whole population including 25 patients with probable sinonasal sarcoidosis (i.e. no histology available).

n.a., not available.

In a recent case control stuy of 20 patients with sinonasal sarcoidosis Aubart et al. showed that sinonasal involvement preceded the diagnosis of sarcoidosis in the majority of the patients [18], which is not in line with our observation. In our case series the majority of patients developed sinonasal involvement after the initial diagnosis of sarcoidosis.

CT scans of the paranasal sinuses were done in 11 of 12 patients. In our patients we noticed a predominance of both maxillary sinuses. CT findings of mucosal thickening and sinus opacification were seen frequently in our patients, whereas other signs of chronic sinus inflammation like destruction, erosions or bone lesions were not seen, but are described in the literature [7,19]. Granulomas or nodules are not always visible and constitute a frequent and helpful diagnostic criterium of sarcoidosis [7]. Interpretation of other CT features such as opacification of paranasal sinuses, nasal cavities or involvement of nasopharynx depend on the clinical context and are nonspecific radiologic features.

Treatment of sinonasal sarcoidosis includes topical or systemic steroids and sinonasal surgery [7,11,12,19]. Response to treatment varies from complete remission to no improvement at all [7]. All of our patients received systemic corticosteroid treatment. In some cases the indication for systemic therapy with corticosteroids was driven by other organ involvements as well. We had one patient, who had to undergo repeated sinus surgeries for sinonasal sarcoidosis despite systemic and topical steroid treatment, which indicates that sinonasal sarcoidosis is not clinically trivial but a rather serious complication of sarcoidosis. Relapse of sinonasal sarcoidosis and the need for longterm systemic corticosteroid treatment has been encountered as a clinical problem in case series before [12].

Interestingly, all of our patients with sinonasal sarcoidosis had pulmonary and lymph node involvement. Furthermore, 8 of 12 patients had one or more additional organ involvement of sarcoidosis. This is in line with recent data demonstrating that multiorgan disease is common among patients with sinonasal sarcoidosis [4,7,12].

Pulmonary involvement according to the chest-X-ray-grade of Scadding [14] was graded as type I and II in our patients. This is consistent with the relatively mild impairment of lung function in the majority of our patients.

## Conclusion

To conclude sinonasal involvement is a rare disease manifestation of sarcoidosis. The key to the diagnosis of sinonasal involvement in sarcoidosis is the clinical awareness of this disease manifestation. Biopsy with histopathological examination needs to be performed to confirm the diagnosis. The clinical course of sinonasal sarcoidosis can be complicated by relapse despite longterm systemic immunosuppressive treatment and repeated sinus surgery.

## Abbreviations

ACE: Angiotensin-converting enzyme; DLCO: Diffusion capacity of the lung for carbon monoxide; ENT: Ear nose throat; CT: Computed tomography.

## Competing interests

The authors declare that they have no competing interests.

## Authors’ contributions

DK initiated and supervised the project. All authors participated in the writing of the manuscript and approved its submission.

## Pre-publication history

The pre-publication history for this paper can be accessed here:

http://www.biomedcentral.com/1471-2466/13/59/prepub
